# Heavy Metal Pollution in Xinfengjiang River Sediment and the Response of Fish Species Abundance to Heavy Metal Concentrations

**DOI:** 10.3390/ijerph191711087

**Published:** 2022-09-04

**Authors:** Guoxiu Shang, Xiaogang Wang, Long Zhu, Shan Liu, Hongze Li, Zhe Wang, Biao Wang, Zhengxian Zhang

**Affiliations:** 1 Key Laboratory of Navigation Structure Construction Technology of Ministry of Transport, Nanjing Hydraulic Research Institute, Nanjing 210029, China; 2 Laluo Hydro-junction and Irrigation District Administration of Tibet Autonomous Region, Lhasa 851414, China

**Keywords:** heavy metal, sediment, ecological risk, fish species

## Abstract

Xinfengjiang River, the largest tributary of Dongjiang River, plays a key role in the water supply of Heyuan, Huizhou, Guangzhou and even the Pearl River urban agglomeration. It is crucial to determine the pollution status, potential ecological risk degree of heavy metals in Xinfengjiang river sediment and their influence on the abundance of fish species. In this paper, seven heavy metal concentrations in sediment from the Heyuan section of the Xinfengjiang river were investigated. The order of average concentration was: As > Zn > Pb > Cr > Cu > Cd > Hg. The average concentrations of Cd, Zn and Cu in the upper reaches of the Xinfengjiang Reservoir were significantly higher than those in the reservoir. The mean value order of Igeo was: Cd > Zn > Pb > As > Cu > Cr > Hg. Cd and As had the highest ecological risk index and the greatest threat to the ecological environment. Pearson correlation analysis and principal component analysis demonstrated that the pollution source of heavy metals such as Cu and Cd are much more likely to originate from the mine fields located in the northeast of the sampling sites. In addition, agriculture, electronic industry and domestic sewage also contributed to the concentration of heavy metals in different degrees. Redundancy analysis showed that the abundance of Cypriniformes was negatively correlated with Cu and Cd concentrations, suggesting that mining activities might indirectly affect the abundance of fish species.

## 1. Introduction

Heavy metals have received widespread attention in the past few decades due to them being persistent hazards to aquatic ecosystems and human health, which are constantly released by natural and anthropic activities, such as mining, industrial and domestic sewage discharge, agriculture, e-waste, soil erosion as well as rock weathering [1,2,3,4]. As one of the components of an aquatic ecosystem, sediment plays an important role as both a concentrated sink of heavy metal pollution, and to some extent an inevitable source [5,6]. A significant amount of heavy metals entering water are deposited at the bottom of the water body with suspended particles, and when the physical and chemical factors (such as pH, oxidation–reduction potential and organic matter) change, they can be released again to cause secondary pollution [7,8,9].

The toxic effects of heavy metals could disturb the metabolism and reproduction of living organisms, cause the death of organisms and affect the aquatic biological community through bio-accumulation mechanism [10,11]. The essential micronutrients such as Cu, Zn, Cr, Mn and Fe may induce toxic effects on organisms at high concentration levels of exposure, while non-essential metals such as Cd, Pb, As, Ni and Hg enhance the overall toxic effects even at low concentration levels [12]. In addition, Pb, Cd and Hg do not perform any known functions in human biochemistry or physiology, nor do they naturally exist in organisms [13]. There is extensive literature on the accumulation of heavy metals in fish [3,10,11,14,15,16]. Cd, As, Cu, Zn, Pb and Cr have toxic effects to C. carpio which are sensitive to heavy metals concentration [17]; Cu, Zn, Cd and Pb in particular have the highest bioaccumulation in C. carpio [18].

Mining operations are considered the greatest threat to ecological integrity because of the lasting toxicity (hundreds of years after the cessation of mining operations) [19,20]. Furthermore, the discharge of acid mine drainage and mining tailings are mainly associated with the pollution of heavy metals in water and sediment [21]. Iron-ore extraction is typically performed through open pit mining, which offers higher productivity and lower costs and security risks compared to underground mining [22]. 

Xinfengjiang River is the largest tributary of Dongjiang River which serves about 28 million people by lying at the heart of drinking water supply source for Heyuan, Huizhou, part of Dongguan, Shenzhen and Hong Kong [23]. It is closely related to the survival and sustainable development of the urban agglomeration in the east of the Pearl River Delta [24]. Xinfengjiang River Basin has a long history of mining [23,25]; the river lies in a metallogenic belt rich in metals such as W, Sn, Zn and Pb. Many illegal small-scale mining sites are widely distributed in the middle reaches of the river, and there are several large-scale sources of mineral input including the Dading iron mine and the Jubankeng tungsten mine. In addition, the non-point source pollution caused by agricultural chemical fertilizers and pesticides is worthy of attention [26], and the electronic industry pollution caused by industrial parks is generally high. Jinfeng Chen et al. [23] determined the content of trace elements in the riverbed, bankside and adjacent agricultural soil of the Zhongxing River which is located in the Dongjiang River Basin. Yun-jiang Yu et al. [27] evaluated the heavy metals and pollution levels in the sediments of the Xinfengjiang Reservoir and the Dongjiang River Basin. Few researchers have addressed the problem of response of fish species to heavy metal pollution in the Xinfengjiang River. Fish are considered to be the most significant biomonitors of aquatic systems for the evaluation of heavy metal pollution levels [28]. This paper focuses on the heavy metal pollution conditions in the sediment of the Xinfengjiang River in the Heyuan city section. Geo-accumulation index, individual heavy metal ecological risk index ($E_{r}^{i}$) and potential ecological risk index (RI) were used to assess ecological risk levels. We then traced the main sources of heavy metals by Pearson correlation analysis and principal component analysis (PCA). Finally, redundancy analysis (RCA) was used to research the response relationship between fish species and heavy metals concentration in sediment.

## 2. Materials and Methods

### 2.1. Study Area

**Figure 1:**
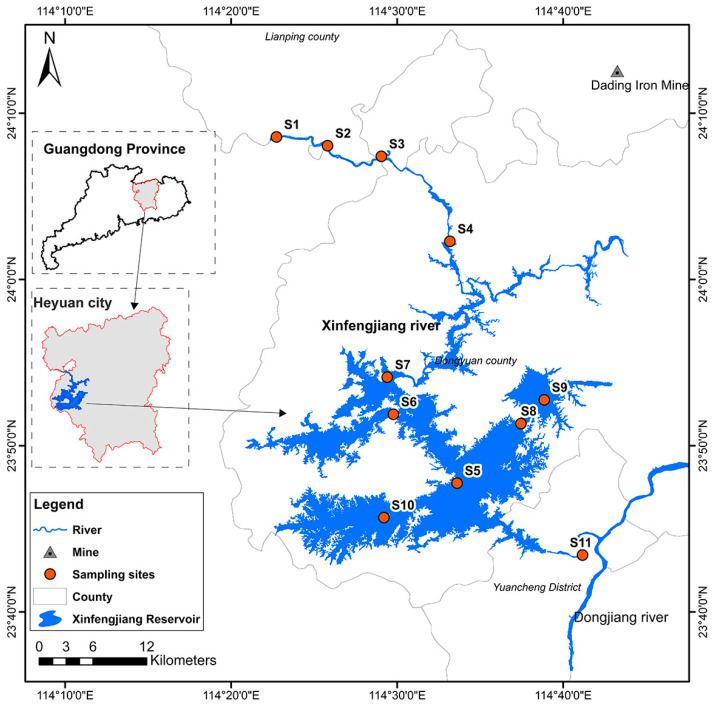
Study area and the location of the sampling sites.

The Xinfengjiang River, the largest tributary of the Dongjiang River, is located in the Northeast of the Guangdong Province (Figure 1). The Xinfengjiang river is 163 km long, of which 94.16 km are in Heyuan City. The Xinfengjiang Reservoir, also known as Lvwan Lake, is the largest artificial reservoir in the Guangdong Province. The study area (23.7° N–24.4° N to 114.2° E–114.7° E) was located in Heyuan City in the middle and lower reaches of the Xinfengjiang river, accounting for 57.8% of the total length of the Xinfengjiang river, with a drainage area of 4340 km, accounting for 74.5% of the total basin area (Figure 1). Influenced by maritime climate, it belongs to a subtropical monsoon climate zone. The average temperature is 19.5~20.7 °C and the average annual precipitation is more than 1500 mm. Eleven sampling sites (S1—S11) were set up in the study area, of which four (S1–S4) were located in the upper reaches of Xinfengjiang reservoir, one (S11) in the lower reaches of the reservoir adjacent to the Dongjiang River and the other six sampling sites (S5–S10) in the reservoir (Figure 1). On the basis of consulting the research literature on heavy metals and examining the actual situation in the study area, seven target heavy metals, Cu, As, Pb, Zn, Cr, As and Hg, were selected for assessment.

### 2.2. Sample Collection and Analysis

#### 2.2.1. Sediment Sampling

Sediment samples (0–10 cm depth) were collected at the corresponding sampling sites in August, 2021. We received a portable GPS device for recording geographic location. Three samples were collected at each site using a portable Ekman grab sampler and subsequently mixed, then placed in an acid-rinsed polyethylene plastic bag and sealed. The sediment samples were transferred to the laboratory for treatment while being kept at 4 °C. 

#### 2.2.2. Sediment Sample Analysis

A standard reference and a reagent blank were included in the heavy metal concentration test to ensure data accuracy and precision. After natural air drying in the laboratory at room temperature, gravel and plant roots in the samples were removed, ground and sifted through 100 mesh for the analysis of heavy metals, including Cu, Cd, Zn, Pb, Cr, As and Hg. The samples were digested with a microwave digestion instrument (CEM Inc., Matthews, NC, USA) for 10 h before concentration analysis. SK-2003AZ atomic fluorescence spectrophotometer(Suokun technology, Beijing, China) was used to analyze the content of As and Hg, while the other five Heavy metal concentrations were analyzed by WFX-120 atomic absorption spectrophotometer(Beifen-Ruili, Beijing, China). Each heavy metal concentration was tested three times, and the data were unacceptable until the relative standard deviation <5%.

#### 2.2.3. Fish Sampling

Fish collection was carried out from August to September in 2021 by laying drift Gill net and ground cage, and the collection points was the same as sediment. Each drift Gill net is 50 m in length and 2 m in height, with a mesh of 3 cm. The statistical catch was recovered after placing a drift Gill net and a ground cage at each station for one night. The fish specimens collected from various survey sites were photographed and weighed at the scene, then tagged and brought back to the laboratory for identification, analysis and preservation. 

### 2.3. Assessment of Heavy Metal Concentration in Sediments

#### 2.3.1. Geo-Accumulation Index (Igeo)

The geo-accumulation index (Igeo) is one of the most widely used and simple indices to evaluate heavy metal pollution in sediment on account of its ability to reflect the enrichment situations and to provide consistent values for comparison [29]. It was calculated using the following empirical relations [30]:

Igeo = log_2_(C_n_/1.5B_n_) (1)

where C_n_ is the concentration of study metal and B_n_ is the background concentration, as listed in Table 1 Factor 1.5 was used for the background matrix correction and reducing lithogenic effects. Table 2 showed the Igeo classes and corresponding sediment quality.

#### 2.3.2. Potential Ecological Risk Index (RI)

The potential ecological risk index is used to assess the toxicity of metal in sediments [25,32,33]. $E_{r}^{i}$ and RI represent individual heavy metal ecological risk and comprehensive potential ecological risk, respectively. Their calculation formulas are as follows:

(2) $$E_{r}^{i}=T_{r}^{i}\times \left(\frac{C_{i}}{C_{0}}\right)$$

(3) $$RI=\sum_{i=1}^{n}E_{r}^{i}=\sum_{i=1}^{n}T_{r}^{i}\left(\frac{C_{i}}{C_{0}}\right)$$

where C_i_ is the concentration of metal i in sediment, C_0_ is the background concentration of metal, and $T_{r}^{i}$ defines biological toxicity factor for individual metal. The $E_{r}^{i}$ values for Cu = Pb = Ni = 5, Zn = 1, Cr = 2 and Cd = 30 [30,31], Table 2 gives the classes and levels for $E_{r}^{i}$ and RI.

### 2.4. Statistical Analysis

Principal component analysis (PCA) was performed to extract significant PCs and associated loads. Pearson’s correlation analysis was used to test the significant relationship between variables. Redundancy analysis (RCA) was used to analyze the response of fish abundance to heavy metal content using Canoco5.0 (Microcomputer Power, Ithaca, NY, USA). PCA and correlation analysis were implemented with R language and IBM Spss26.0.

## 3. Results

### 3.1. Heavy Metal Concentration in Sediments

The concentrations of seven heavy metals in surface sediments of Xinfengjiang River (Heyuan section) was shown in Table 1. The mean concentrations followed a decreasing ranking order of As (86.38 mg/kg) > Zn (85.13 mg/kg) > Pb (62.75 mg/kg) > Cr (35.04 mg/kg) >Cu (28.53 mg/kg) >Cd (0.602 mg/kg) >Hg (0.078 mg/kg). The variable coefficients of As, Cu and Cd concentrations with values of 172%, 141.6% and 129.1%, respectively, were higher than other four heavy metals, in other words, it is more reasonable to believe that the sources of these three heavy metals are more closely related to the effects of human activities. There was significant difference in the concentrations of most heavy metals among upstream sites (S1–S4), reservoir sites (S5–S10) and the downstream site (S11). The heavy metal contents were generally higher in the upstream than reservoir, while Hg content shows the highest values at the downstream site (S11). Particularly, S4 was the highest total heavy metals concentration. 

### 3.2. Igeo

**Figure 2:**
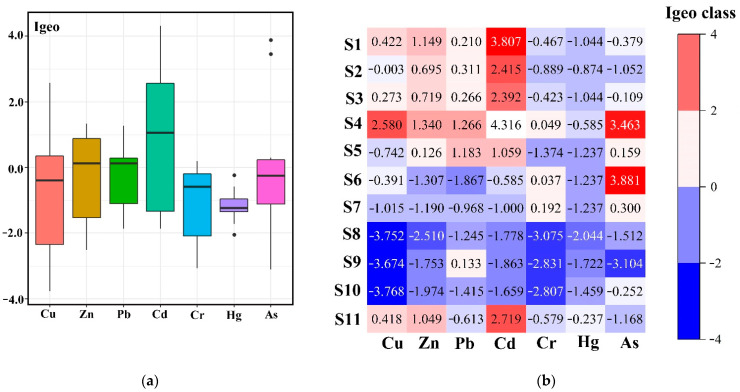
Geo-accumulation index (Igeo) of heavy metals in sediment: (**a**) Boxplot of Igeo values; (**b**) Igeo class in different sites.

The distribution of Igeo value of heavy metals in the sediments is shown in Figure 2. The maximum Igeo values of Hg and Cr were below 0 and 1, respectively, indicating that the sediments were not contaminated with these two metals, or that the contamination level was low. The Igeo values of Cu (−3.76–2.58, median −0.39), Zn (−2.51–1.34, median 0.13), Pb (−1.87–1.27, median 0.13), As (−1.51–3.88, median −0.25), Cd (−1.86–4.32, median 1.06). Different colors (Figure 2b) refer to different levels of sediment pollution, with blue indicating uncontaminated, and from light yellow to orange and red indicating slightly contaminated to extremely contaminated. Cd exceeded the background value most seriously, with the highest Igeo value exceeding 4, from heavily to extremely contaminated; Cu, Zn, Pb and Cd concentrations at four sites (S1–S4) upstream of the reservoir all exceeded the regional background values. As appeared in extremely high values at points S4 and S6, exceeding other sites by a large margin. Pollution is more severe upstream (S1–S4) and downstream (S11) than at the Reservoir (S5–S10).

### 3.3. Ecological Risks and Potential Ecological Risks

**Figure 3:**
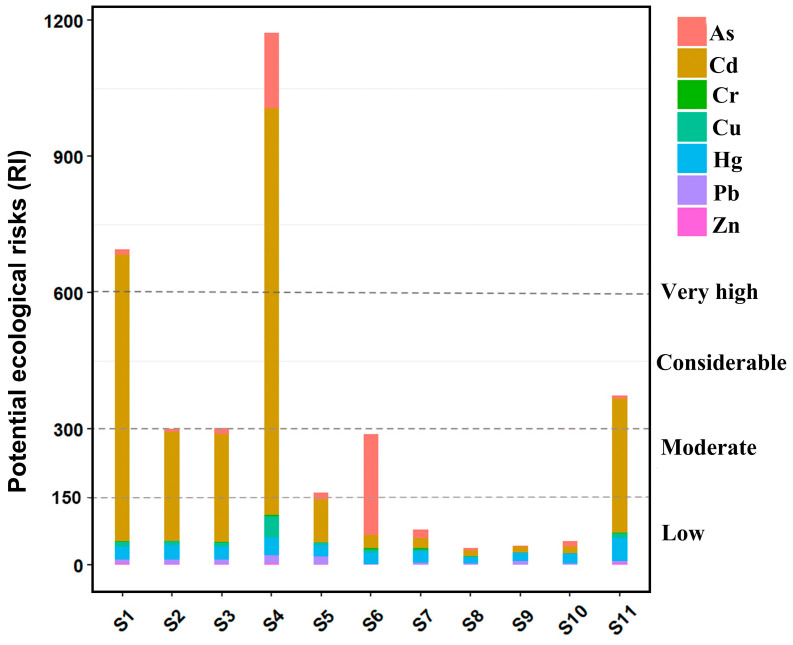
RI value of heavy metals in sediment (derived from the $E_{r}^{i}$ value).

The single metal ecological risk index ($E_{r}^{i}$) and potential ecological risk index (RI) of heavy metals in the sediments of Heyuan City section of Xinfengjiang river are shown in Table 3 and Figure 3. Zn, Pb and Cr at the low ecological risk with individual ecological risk indices lower than 40; Cu showed moderate ecological risk at S4, Hg was in the threshold of low risk (Er = 40) at S4 and moderate risk at S11; high ecological risk at S4 and S6, and the above three total metals were of low risk at the rest of the sites. Cd created the highest degree of ecological risk with six sample sites reaching moderate risk or higher, and the biggest two ecological risk values of were 630 (S1) and 896.25 (S4). The potential ecological risk indices of the sampling sites ranked in order as follow: S4 > S1 > S11 > S3 > S2 > S6 > S5 > S7 > S10 > S9 > S8, consistent with above, S4 and S1 had the highest RI, 1171.4 at S4 and 694.8 at S1, all above the high potential risk limit of 600. S7, S10, S9 and S8 were sampled in the low potential risk range. In summary, Cd and As posed the greatest threat to ecology, followed by Hg, Cu, Zn and Cr. As with the results of Igeo, the ecological risk of the four upstream sites were higher than the reservoir sites, and the downstream was in the middle. S4 was the most prominent, S5 and S6 were observed to significantly higher than other sites in reservoir.

### 3.4. Tracing the Sources of Heavy Metal Pollution

Pearson correlation analysis and principal component analysis are widely used to explore the internal correlation of variables and help trace the pollution sources of heavy metals in sediments [34,35]. Table 4 listed the Pearson correlation matrix among sediment heavy metals. All metals showed positive correlations, with Cu-Cd (r = 0.883), Cd-Zn (r = 0.891) and Zn-Hg (0.804) showing highly significant positive correlations at *p* < 0.01, and Cu-Zn (r = 0.723), Cu-Pb (r = 0.643), Cd-Pb (r = 0.61) and Hg-Cd (r = 0.602) showing significant positive correlation at *p* < 0.05. Statistically significant heavy metals with high correlation coefficients are considered to have the same origin or similar behavior during river transport [36]. Lack of valid correlation among the other metals reveals that the contents of these metals are not controlled by a single factor.

**Figure 4:**
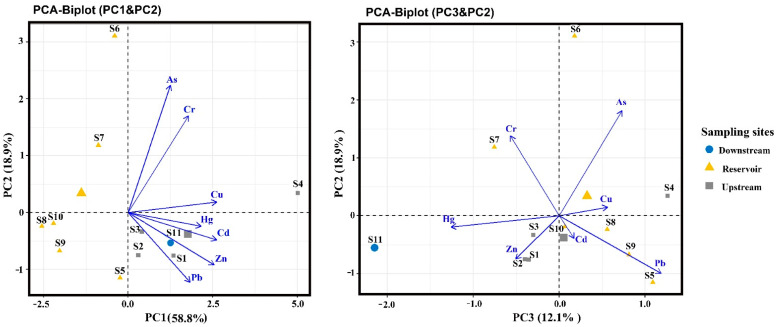
Principal component analysis (PCA) of heavy metals in sediment sampling sites.

Principal component analysis (PCA) of heavy metals was given in Figure 4. The first principal component explained 58.8% of the variance, the second principal component explained 18.9%, and the three principal component axes together explained 89.8% of the overall variance. The loads of each heavy metal on the three components are shown in Table 5.

Cu, Cd and Zn have obvious advantages on PC1, loading 0.926, 0.923 and 0.898 respectively. Different from the former, Hg, Pb and Cr also have varying degrees of loading on the other two principal components, although their loadings on the first principal component are not low (0.762, 0.647 and 0.632). Among them, Hg loads −0.549 onPC3, Pb loads −0.432 on PC2 and 0.518 on PC3, Cr loads 0.603 on PC2. It is thus clear that the sources of these three elements are significantly different from the previous three. Meanwhile, it is worth noting that As differs from the previous heavy metals in that its loading on PC2 (0.792) has a significant upper hand. In the PCA-Biplot (PC1 and PC2) (Figure 4), the reservoir sites (S5 to S10) are all distributed in the third and fourth quadrants, the upstream site S4 near the reservoir is distributed in the first quadrant, and the remaining three upstream sites (S1–S3) and one downstream site (S11) are distributed in the second quadrant. S4 and S6 are outliers, with S4 closer to PC1 and S6 closer to PC2. In the PCA-Biplot (PC3 and PC2) plot (Figure 4), S4 is closer to the positive direction of the third principal component and point S11 is closer to the negative direction of the third principal component. This indicates that the content of heavy metals in the upstream and downstream sites is higher than that in the reservoir sites, and the content of Hg at S11 is much higher than others, which is consistent with the results reflected by the ecological risk index of sampling sites.

### 3.5. Response of Fish Species Abundance to Heavy Metal Concentration in Sediment

**Figure 5:**
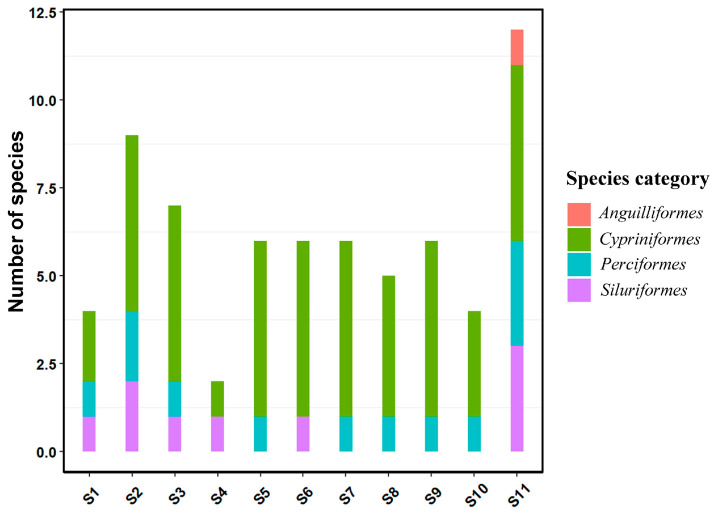
Distribution of fish species at sampling sites.

A total of 23 species of fishes belonging to four orders were recorded in this survey, which were cypriniformes, perciformes, cypriniformes and siluriformes, all of them belonged to teleost. Cypriniformes are dominant at the order level, as shown in Figure 5.

**Figure 6:**
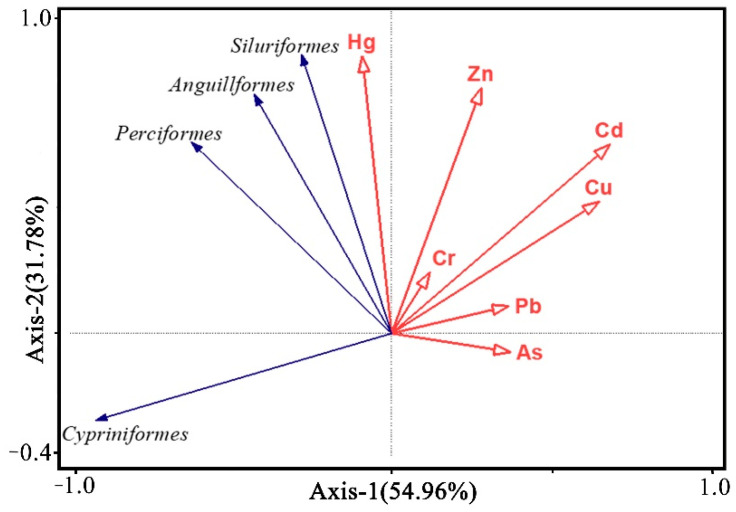
Redundancy analysis diagram between heavy metals and fish species.

It has been reported that heavy metals in the environment will be enriched in fish, and there is a response relationship between the abundance of fish species and heavy metals in the environment, which affects the composition and abundance of organisms to varying degrees [37]. Redundancy analysis is a ranking method combining regression analysis with principal component analysis, which can intuitively reflect the response of species variables to environmental variables [38]. The magnitude and angle of vectors can intuitively reflect the correlation between variables. The first axis explains 54.96% of the species abundance variables, and the two axes jointly explain 86.74% of Figure 6.

## 4. Discussion

Both the geo-accumulation index and the ecological risk index demonstrate that the sediment heavy metals pollution degree in the upstream sites were generally higher than that in the reservoir area, and the downstream site was in the middle. Among them, Cd, Cu, As and Zn were the main heavy metal pollutants. They could be caused by a variety of reasons. First and foremost, the large mining sites are located in the northeast of the upstream site. Second, the upstream is closer to the community. The upstream content of heavy metal pollutants produced by agricultural chemical fertilizers and pesticides, domestic sewage discharge, breeding, etc., is also higher than that in the reservoir area. In view of the industrial characteristics of the Lianping and Dongyuan counties around the Xinfengjiang Reservoir, it was speculated that the first principal component in the principal component analysis (Figure 4) was mainly related to mineral exploitation, livestock and poultry breedin and agricultural activities. The river lies in a metallogenic belt in the northeast Guangdong province which is rich in metals such as W, Sn, Pb, Fe and Zn [39]. There are many scattered illegal small-scale mining sites as well as large and medium mining sites in the upper reaches of the reservoir, so mining activities are prevalent in this area [23]. The Lianping County is rich in mineral resources, with more than 30 kinds of proven minerals and approximately 52 mining locations. Iron ore has the greatest advantage with a reserve of 160 million tons. The Jubankeng tungsten mine, located in Lianping County, is the largest tungsten mine that has been operating intermittently since last century in the Guangdong Province. Located in the Lianping County, the Sawpan Hang tungsten mine, which mines tungsten and copper ore, is the largest tungsten mine in the Guangdong Province and has been operating intermittently since the last century, about 45 km from the upstream sampling point of the reservoir. Dading Iron Mine, located in Youxi Town, Lianping County, is the largest iron mine in the province, 25.5 km away from S4. Previous studies have shown that tailings, drainage and air suspended solids caused by mining activities will strongly affect the content of heavy metals in nearby water, sediment and soil [22]. In particular, the content of heavy metals such as Cd, Cu, Zn and Pb is generally higher than the background value, and can be up to a thousand times in Pilcomayo river [40]. Many studies confirmed the migration of trace elements from point source to downstream with sediments, and demonstrated that the contents of trace elements in downstream rivers generally showed a decreasing trend [41]. In this paper, the average concentration of the upstream points (S1–S4) of Cd, Cu and Zn is much higher than that of the reservoir points (S5–S10), which are 16 times, 6.9 times and 4.5 times higher, respectively.

According to investigation, pig farming contributes to the concentration of Cu in sediments, especially the use of copper-containing additives in pig feed, which results in high levels of copper in pig manure [42]. There are a large number of pig farms in the Xinfengjiang catchment area; on the one hand, despite sewage treatment, some of the copper and cadmium in pig manure is discharged into rivers along with the wastewater. On the other hand, pig manure, as the main source of manure, enters the soil with agricultural activities, indirectly increasing the content of heavy metals in the soil, and some of the heavy metals in the soil will be discharged into the river with the runoff. Cd and Zn have strong fluidity. Cd is a symbolic metal in pesticides and fertilizers involved in agricultural activities [43]. Besides mining, gasoline, phosphate and domestic sewage are also possible sources of Cd [44]. The second principal component may mainly represent natural erosion processes such as parent rock weathering and soil erosion. The third main component mainly associates with industrial activities, electronics, machinery manufacturing and domestic sewage discharge. There are five outfalls distributed near S11 downstream of the Xinfengjiang Reservoir Dam; meanwhile, the downstream of the reservoir converges into the Dongjiang River, in close proximity to industrial parks such as the Linjiang Industrial Zone and Zijin Industrial Park, which mainly produce electronics and electrical machinery [27]. The main pollutants are Cr, As, Cu and Pb. As is widely used in semiconductors, diodes, alloys and structural steel. Pb is the raw material of battery, and lead compounds are widely used in pigment, glass, plastic and rubber. Meanwhile, Pb is used in various metallurgical equipment and chemical manufacturing. Hg mainly comes from domestic sewage, manufacturing industry and electronic appliances.

Overall, the heavy metals in the Xinfengjiang river sediment are from a wide range of sources, including mining, agricultural activities, industrial activities, domestic sewage and rock weathering. Cu may originate from mining, farming and pesticide herbicides. Cd is correlated with mining, farming, industrial production, domestic sewage, agricultural activities, etc. Pb is mainly correlated with automobile exhaust pollution, sewage and electronic appliance pollution. As may be correlated with pesticides, Hg is mainly from domestic sewage and industrial activities.

In addition, heavy metal pollution can negatively affect fish species abundance. Cypriniformes are negatively correlated with heavy metals except Hg, and significantly negatively correlated with Cd and Cu. Perciformes are negatively correlated with As and Pb. Hg is positively correlated with Perciformes, Silphiformes and Anguilliformes, but not with Cypriniformes. Therefore, Cu and Cd have a great influence on the species of Cypriniformes, and high concentrations are detrimental to species abundance. In fact, Hg is considered as one of the most toxic elements or substances on earth. Research indicates that Hg exposure can induce various adverse effects on fish at physiological, histological, biochemical, enzymatic and genetic levels [45]. As a result of redundancy analysis, there are five sewage outlets in the section from the Xinfengjiang Reservoir Dam to the Dongjiang River, and sewage may be the biggest contributor of mercury. The abundance of fish at S11 is the highest among all points, mainly because it is close to the Dongjiang River, rather than Hg having a positive impact on the abundance of fish species. Moreover, water flow velocity is one of the important influencing factors in the living environment of fish, which in upstream points (S1–S4) is higher than that in the reservoir area (S5–S10), and the above redundancy analysis result shows Cd and Cu have a higher degree of negative influence on the Cypriniformes, indicating that the mobility of these two elements and the difference between the upstream and the reservoir area are stronger. The abundance of Cypriniformes in the upstream points was quite different, and the mean value was small. It indicates that the flow velocity has an adverse effect on the Cypriniformes, and this conclusion is consistent with the effect of heavy metals. That is to say, the flow velocity also has influence on the distribution of heavy metals such as Cd and Cu in the sediment, thereby affecting the response of fish species abundance to the concentration of heavy metals.

## 5. Conclusions

The pollution and toxicity of heavy metals in sediments is one of the most concerning issues in aquatic ecosystems. This study examines seven heavy metals (Cu, Zn, Pb, Cd, Cr, Hg and As) in the sediment at 11 sampling sites located in the Heyuan section of the Xinfengjiang River. Based on the results related to heavy metals and their analysis, the following conclusions could be drawn: (1) The concentrations of heavy metals and ecological risks in the upstream of the Xinfengjiang Reservoir are generally higher than those in the reservoir, and the downstream is in the middle. (2) The mean order of Igeo was: Cd>Zn>Pb>As>Cu>Cr>Hg, and the Cd at S1 and S4 reached a heavily contaminated level. The potential ecological risk index showed that Cd and As were the most serious heavy metals threatening the ecology. The order of potential risk index on sampling sites was: S4 > S1 > S11 > S3 > S2 > S6 > S5 > S7 > S10 > S9 > S8. (3) Pearson correlation analysis and principal component analysis showed that Cd, Cu and Zn had a high correlation, and Hg and Zn had a very significant correlation. The possible sources of heavy metals mainly include mining, agricultural activities, industrial activities and domestic sewage. Mine pollution should be given due attention. (4) Redundancy analysis was used to analyze the response of fish species abundance to heavy metal concentrations, and the results showed that the Cypriniformes was most affected by Cd and Cu. It is speculated that mining is the main source of heavy metals in Xinfengjiang River sediment, and indirectly affects fish species abundance. Water flow velocity is an influencing factor of fish species abundance, and may even be the main factor. In this regard, this paper is still insufficient, and it is expected that this issue can be further studied in the future.

## Figures and Tables

**Table 1:** Statistics of heavy metal concentration in sediments of XFJR.

Sample Sites	Cu (mg/kg)	Zn (mg/kg)	Pb (mg/kg)	Cd (mg/kg)	Cr (mg/kg)	Hg (mg/kg)	As (mg/kg)
S1	32.50	163.0	68.8	1.680	41.80	0.08	22.80
S2	24.20	119.0	73.8	0.640	31.20	0.09	14.30
S3	29.30	121.0	71.5	0.630	43.10	0.08	27.50
S4	145.00	186.0	143.0	2.390	59.80	0.11	327.00
S5	14.50	80.2	135.0	0.250	22.30	0.07	33.10
S6	18.50	29.7	16.3	0.080	59.30	0.07	437.00
S7	12.00	32.2	30.4	0.060	66.00	0.07	36.50
S8	1.80	12.9	25.1	0.035	6.86	0.04	10.40
S9	1.90	21.8	65.2	0.033	8.12	0.05	3.45
S10	1.78	18.7	22.3	0.038	8.26	0.06	24.90
S11	32.40	152.0	38.9	0.790	38.70	0.14	13.20
Mean	28.50	85.13	62.75	0.602	35.04	0.078	86.38
C_V_(%)	141.60%	76.70%	69%	129.10%	61.90%	35.60%	172%
Background value	16.17	48.99	39.65	0.08	38.53	0.11	19.77

**Table 2:** Classes of contamination indices and corresponding levels.

Igeo Class ^a^	Sediment Quality	$E_{r}^{i}\;\mathrm{Class}{\;}^{b}$	Potential Risk	RI Class ^c^	Ecological Risk
<0	Uncontaminated	$E_{r}^{i}<40$	Low	RI<150	Low
0–1	Uncontaminated to moderately contaminated	$40\leq E_{r}^{i}<80$	Moderate	150≤RI<300	Moderate
1–2	Moderately contaminated	$80\leq E_{r}^{i}<160$	Considerable	300≤RI<600	Considerable
2–3 3–4 4–5	Moderately to heavily contaminated Heavily contaminated Heavily to extremely contaminated	$160\leq E_{r}^{i}<320$ $E_{r}^{i}\geq 320$	High Very high	RI≥600	Very high
>5	Extremely contaminated				

Igeo the geo-accumulation index, $E_{r}^{i}$ the potential ecological risk factor of single metal, RI the potential ecological risk index, CF the contamination factor, PLI the pollution load index. ^a^ [30]; ^b^ [31]; ^c^ [32].

**Table 3:** Individual ecological risks ($E_{r}^{i}$) and potential ecological risks (RI) of heavy metals.

Sites	$E_{r}^{i}$							RI
	Cu	Zn	Pb	Cd	Cr	Hg	As	
S1	10.05	3.33	8.68	630.00	2.17	29.09	11.53	694.8
S2	7.48	2.43	9.31	240.00	1.62	32.73	7.23	300.8
S3	9.06	2.47	9.02	236.25	2.24	29.09	13.91	302.0
S4	44.84	3.80	18.03	896.25	3.10	40.00	165.40	1171.4
S5	4.48	1.64	17.02	93.75	1.16	25.45	16.74	160.2
S6	5.72	0.61	2.06	30.00	3.08	25.45	221.04	288.0
S7	3.71	0.66	3.83	22.50	3.43	25.45	18.46	78.0
S8	0.56	0.26	3.17	13.13	0.36	14.55	5.26	37.3
S9	0.59	0.44	8.22	12.38	0.42	18.18	1.75	42.0
S10	0.55	0.38	2.81	14.25	0.43	21.82	12.59	52.8
S11	10.02	3.10	4.91	296.25	2.00	50.91	6.68	373.9

**Table 4:** Pearson’s correlation matrix of sediment heavy metals.

Heavy Metals	Cu	Zn	Pb	Cd	Cr	Hg	As
Cu	1						
Zn	0.723 *	1					
Pb	0.643 *	0.596	1				
Cd	0.883 **	0.891 **	0.610 *	1			
Cr	0.520	0.426	0.106	0.438	1		
Hg	0.577	0.804 **	0.281	0.602 *	0.492	1	
As	0.528	0.084	0.093	0.287	0.582	0.154	1

* Correlation is significant at the 0.05 level (2-tailed). ** Correlation is significant at the 0.01 level (2-tailed).

**Table 5:** The interpretation variance of heavy metals on three principal components.

Heavy Metals	PC1	PC2	PC3
Cu	0.926	0.066	0.246
Cd	0.923	−0.169	0.076
Zn	0.898	−0.325	−0.220
Hg	0.762	−0.085	−0.549
Pb	0.647	−0.432	0.518
Cr	0.632	0.603	−0.248
As	0.442	0.792	0.319

## Data Availability

Not applicable.
